# The Effect of Cardiopulmonary Resuscitation and Cardiac Chest Pain Management Training on Perceived Control, Depression, Stress and Anxiety in the Spouses of the Patients with Myocardial Infarction: A Randomized Controlled Trial

**DOI:** 10.30476/IJCBNM.2020.81315.0

**Published:** 2020-04

**Authors:** Fatemeh Afrasiabi, Zahra Molazem, Arash Mani, Alireza Abdi Ardekani

**Affiliations:** 1 Student Research Committee, School of Nursing and Midwifery, Shiraz University of Medical Sciences, Shiraz, Iran; 2 Community Based Psychiatric Care Research Center, School of Nursing and Midwifery, Shiraz University of Medical Sciences, Shiraz, Iran; 3 Research Center for Psychiatry and Behavior Science, Shiraz University of Medical Sciences, Shiraz, Iran; 4 Department of Cardiology, School of Medicine, Shiraz University of Medical Sciences, Shiraz, Iran

**Keywords:** Cardiopulmonary resuscitation, Chest pain, Myocardial infarction, Spouses, Stress

## Abstract

**Background:**

Sudden cardiac death is the most common cause of mortality worldwide. Most cases occur at home and the individuals most likely witnessed are family members. Spouses play a significant role in the recovery of such patients. We aimed to investigate the effect of Cardiopulmonary Resuscitation (CPR) and cardiac chest pain management trainings on the perceived control, depression, stress and anxiety in the spouses of the patients with Myocardial Infarction (MI).

**Methods:**

The present randomized controlled trial study was performed on 78 spouses of the patients admitted to Cardiac Care Unit of the hospitals affiliated to Shiraz University of Medical Sciences from August 2013 to April 2014. The subjects were randomly assigned into intervention (n=40) and control groups (n=38). The intervention group took part in educational workshop on CPR and cardiac chest pain management and followed-up for six weeks. Data were collected using Depression Anxiety Stress (DASS) Scale and Rotter’s internal-external locus of Control Scale. Statistical methods of data analysis included descriptive statistics, Chi-squared test, paired sample t-test, independent sample t-test, one way analysis of covariance, and multivariate analysis of covariance.

**Results:**

There were significant differences between intervention and control groups in perceived control and DASS subscales. After the intervention, perceived control scores reduced from 9.42±3.33 to 8.15±3.65 (P=0.001); depression from 28.85±11.99 to 21.65±8.64 (P=0.001); anxiety from 26.6±9.46 to 20.70±6.29 (P=0.02); and stress from 30.20±10.91 to 24.32±10.23 (P=0.01) in the intervention group.

**Conclusion:**

CPR and cardiac chest pain management trainings could effectively improve the perceived control, depression, anxiety, and stress in the spouses of the patients with MI.

** Trial Registration Number:** IRCT201310128124N2.

## INTRODUCTION

Currently, cardiovascular diseases (CVDs) are among the most common chronic diseases in most parts of the world. CVDs are predicted to be the first leading cause of mortality and morbidity worldwide, accounting for over 17 million deaths annually. ^1^ Moreover, cardiac death is an important clinical and public health issue. ^2 , 3^ Presently, sudden cardiac death (SCD) is the most common cause of mortality worldwide. ^4 , 5^

According to the studies conducted in several countries, it is estimated that every 9 seconds someone in the world dies suddenly and 370.000 to 750.000 of these deaths are caused by cardiac arrest. This rate is also increasing annually. ^6^

Evidence shows that almost 70 to 77% of out-of-hospital deaths and most interventions of cardiac deaths occur at home. However, the survival rate of people afflicted with cardiac arrest at home is lower than those who experience it at public places. ^2 , 7^

Among the family members of the patients with myocardial infarction (MI), their spouses play a significant role in the support and recovery of such patients. The patients’ spouses have commonly reported unpleasant symptoms and feelings such as anxiety, depression, fear, loss of appetite, and inability to concentrate. Such psychological symptoms often last for months after the heart attack. ^8^ Psychological issues of spouses and supporting them is crucial due to some reasons. Firstly, many of the spouses commonly suffer from psychological stresses which last longer than those of the patients. Secondly, the spouses’ stress and, especially depression, might be transferred to the patients with MI and also manifested in them. ^7^ Besides, psychological stresses, anxiety and depression are significantly associated with increased morbidity and mortality in the patients. The spouses’ stress can harm their ability to protect and support their patients. One of the effective key points in enhancing the spouses’ protection ability is helping them to control their stress. Psychological stresses occurring after a serious illness follows decreased sense of perceived control. Perceived control is the individuals’ beliefs in an internal locus, which affects the events and environment, so that they will be able to achieve their desired goals. ^9^ Higher levels of internal perceived control can reduce the spouses’ psychological stress. Such a trait can be modified or reinforced using appropriate interventions such as cardiopulmonary resuscitation (CPR). ^8 , 9^ The actions taken during the initial minutes of an emergency, including basic life support (BLS), are critical for the patient’s survival. ^10^ CPR as a public skill is one of the greatest inventions of medical history and prompt delivery of CPR is a crucial determinant of survival for many victims of sudden cardiac arrest. ^11^

Evidence also shows that learning critical measures to be taken to save the patients’ lives in the case of cardiac arrest is one of the most important needs of the spouses of the patients who have had an MI. CPR training has met such a need effectively. ^8 , 9^ On the other hand, 3/4 of SCDs occur at home and in the presence of the victim’s family. Therefore, the family members are most available and important people to the patient. ^12 , 13^

Besides, receiving CPR from bystanders at the scene increases the survival rate by 2 to 3 times. ^14^ Resuscitation performed by bystanders on patients who are in cardiac arrest and prompt arrival of emergency medical services improves the survival rates and enhances the quality of life in the survivors. ^15 , 16^

In addition, one of the important concerns about saving the patient’s life is the lack of knowledge and sufficient skills in patients and their relatives. ^2 , 13^ In this field, several national and international studies have been conducted. In other countries, the studies have focused on the impact of CPR training on perceived control in spouses, and assessed attitudes towards CPR training in family members of patients. ^2 , 9^ In Iran, the studies published up to now assessed the effect of education of CPR on knowledge and skills of health care team such as nursing staff and physician. ^17 , 18^ However, a study focusing on the effect of CPR and cardiac chest pain management training in the family members of patients has not been done. However, the family members are the most suitable target group to receive such training. 

On the other hand, the findings of a study on the family members’ knowledge and performance in dealing with a patient’s heart attack in Iran showed that the patients’ family and companions take them to emergency department without performing appropriate action for heart attack. The results of this study approved the need for education to community members in Iran in order to raise the level of performance and awareness in confrontation with of a patient’s heart attack. ^19^ Due to the important role of the family and spouses in providing support and care for patients and lack of supportive systems and appropriate group training of caregivers in Iran, interventional research on the inclusion of chest pain management and BLS programs is strongly suggested. Therefore, we aimed to investigate the effect of CPR and cardiac chest pain management training on the perceived control, depression, stress, and anxiety in the spouses of the patients with MI

## MATERIALS AND METHODS

The present randomized controlled trial study was performed on the spouses of all 84 patients admitted to Cardiac Care Unit (CCU) of the hospitals affiliated to Shiraz University of Medical Sciences from August 2013 to April 2014. The sample size was calculated as 42 in each group based on the data of similar studies ^20^ and the following formula using Med Calc statistical software with a power of 95%; α of 0.05; mean difference of 12.56; Z_α_ of 1.96; Z_β_ of 1.64; σ of 15.18; and loss rate of 10%. 

$n=\frac{\left[{\left(Z_{\alpha /2}+Z_{\beta }\right)}^{2}\times 2\delta^{2}\right]}{{\left(\mu_{1}-\mu_{2}\right)}^{2}}$

**Figure 1:**
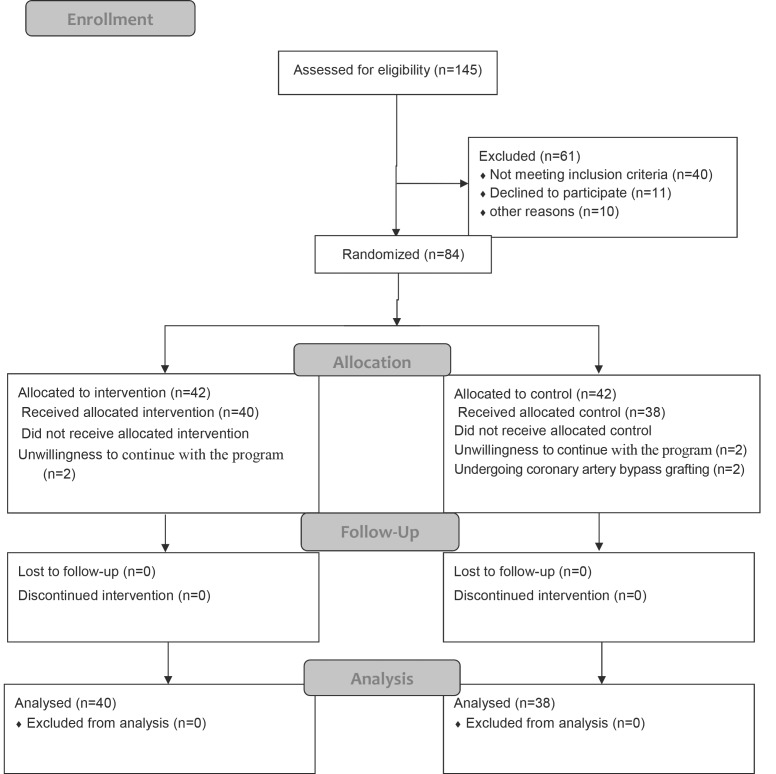
Flowchart of the participants in the randomized clinical trial

A random number table was used to assign 84 participants, who had the inclusion criteria. Inclusion criteria included ability to understand the information presented (which is confirmed by the researcher), ability to participate in research (having reading and writing skills, lack of disability or specific illness), lack of participation in similar training programs simultaneously or since 6 months before the study, age under 65 years old, and willingness to participate in the research. We assigned the individuals to enter numbers 1 to 84 respectively and then use the random number table. We selected 42 numbers from 1 to 84; then, by throwing the coin, we assigned the individuals corresponding to the numbers selected from the random numbers table to the control or intervention groups. 42 participants (from 1 to 84) were also replaced in the other group. Six participants were excluded from the study as they withdrew from participation or had to undergo coronary artery bypass graft. We continued the study with 78 participants in the intervention (N=40) and control (N=38) groups (Figure 1), 40 participants in the intervention group were divided into groups of 6 to 7 participants and each group attended two workshop. The first four-hour workshop included 2 hours of theoretical and 2 hours of individual practical training. The first workshop was initiated by explaining the process of cardiac chest pain management and CPR for the participants in simple words using PowerPoint slides and followed by lectures and group discussions. In the practical session, the participants were trained using CPR torso training manikin (Laerdac Company, France) and all the participants were given an opportunity to practice on the manikin individually. The second workshop was held two weeks later than the first one to review what was taught earlier and all the participants’ questions and ambiguities were answered. Furthermore, a short film about CPR produced based on American Heart Association Guideline 2010 which has been translated into the Persian language was shown for 2 minutes and 42 seconds. To diagnose the chest pain related to MI from other types of chest pain, oral complete explanation was provided and educational pamphlets (including causes of MI, risk factors, pain symptoms, baseline pain, feelings when experiencing MI pain, and pre-hospital actions for managing chest pain and emergency contact if unsuccessful in pain management), were given to the spouses at workshop. Also, they were trained about cardiac arrest (symptoms, causes, stages of cardiopulmonary resuscitation, points to be considered in massage, including breathing and pulse examinations), and a CD of CPR training video was given to them. All the groups were given another chance to fix their problems regarding the practical skills by using the manikin individually. The researcher’s phone number was given to the participant to contact and ask their questions. Moreover, the researcher followed them up for six weeks after the training by telephone and answered their questions about the package and the training. The control group did not receive CPR and chest pain trainings. They only received the CCU’s routine activities including education about the illness and taking medications.

According to Moser et al.’s study on perceived control of spouses, which was based on the follow-up period of 1 month the follow-up period lasted six weeks. ^9^

To measure the rate of stress, anxiety, depression, and perceived control, we administered two questionnaires to the participants of the intervention and control groups before and after six weeks of the research period. Data were collected using Depression, Anxiety and Stress Scale (DASS) and Internal-external Locus of Control scale. DASS is a 42-item self-reported questionnaire designed to measure the severity of depression, anxiety, and stress. Thus, the following cut-off points were developed for defining it. The levels of depression included 0-9 normal, 10-13 mild, 14-20 moderate, 21-27 severe, and 28+ extremely severe. The levels of anxiety included 0-7 normal, 8-9 mild, 10-14 moderate, 15-19 severe, and 20+ extremely severe. Also, the level of stress included 0-14 normal, 15-18 mild, 19-25 moderate, 26-33 severe, and 34+ extremely severe. Each of the three scales contained 14 items and the total score in each scale was calculated by summing the scores for the relevant items. The validity and reliability of DASS were well confirmed by Lovibond and Lovibond. ^21^ Internal consistencies for depression, anxiety, and stress scales of DASS in the normative sample were 0.91, 0.81 and 0.89, respectiely. ^22^

The first three factors together accounted for 41.3% of the item variance. The correlation between the factors were as follows: Depression-Anxiety r=0.42; Anxiety-Stress r=0.46; and Depression-Stress r=0.39 which confirmed the satisfactory reliability of the three scales. The factor structure of DASS was confirmed with two different approaches and findings providing support for the psychometric properties of the DASS scales and their convergent and discriminant validity with other instruments developed on clinical populations. ^22^ Szabo approved the construct validity of DASS. ^23^ In Iran, the reliability of DASS scales was confirmed by examining the coincidence coefficients and test re-test coefficients. In addition, the construct validity of the two scales of depression and anxiety was confirmed by using the correlation coefficient between the scores of the two scales with those of the subjects in the Beck Depression Scale and the four-system anxiety questionnaire. Concurrent validity of sub-scales was determined by calculating the scores of a sub-sample from the general population (n=315) with a group of patients who suffered from psychological disorders. According to the results of this study, DASS scales have the necessary requirements for application in clinical situations with Iranian people. ^24^ In this study, the reliability of this scale was assessed by testing its stability in a group of 30 spouses of the patients suffering from MI from a baseline measurement to six weeks later in the same subjects who received no intervention. The interclass correlation for baseline scores and six week’s scores was 0.96, which confirms the reliability of this scale (alpha=0.96, 0.89 and 0.93 for Depression, Anxiety and Stress). ^25^

Locus of Control scale is designed to measure generalized expectancies for internal versus external locus of control. In this scale, social learning is considered as a theoretical framework. Also, it is a 29-item forced-choice test and each item has two options (A and B). The scale includes six filler items and 23 items scored as 0 or 1. Opposite responses receive no score. It has a minimum score of 0 and a maximum of 23 with the high scores representing an external locus of control and a low score representing an internal locus of control. Rotter describes the development of the tests of individual differences in a generalized belief in internal-external and provides reliability, discriminant validity and normative data for one test, along with a description of the results of several studies of construct validity. The reliability of the Internal Locus of Control Rotter scale has been calculated by the test-retest method; Rotter reported their level from 0.49 to 0.83. Discriminant validity is indicated by the low relationships with such variables as intelligence, social desirability, and political liberalness. Most significant evidence of the construct validity of the Internal-External (I-E) scale comes from the predicted difference in behavior for individuals above and below the median of the scale or from correlations with behavior criteria. ^26^ In Iran, various studies have been conducted on the reliability and validity of the scale. The reliability coefficient of this scale has been calculated 0.70 using two-half-way and Kooder-Richardson method . Also, the reliability coefficient of this scale has been at the same level with a test-retest method within one to two months. The reliability coefficient obtained from the short form of this scale was 0.72 for the students at Noor University, as shown in the Noforsati’s study. ^27^ In the present study, Cronbach’s alpha of Internal Locus of Control scale was 0.75.

This study was approved by the Ethics Committee of Shiraz University of Medical Sciences (IR.SUMS.REC.1392.S6680). The participants were provided with sufficient information about the study procedure and objectives, benefits, nature, and duration of the study. They also signed written informed consents after being ascertained about the confidentiality of their information. They were also ensured that they could withdraw from the study whenever they desired. Statistical methods of data analysis included descriptive statistics (such as mean, number, standard deviation and percentage), Chi-squared test (for demographic variables) , One way analysis of covariance (for perceived control), and multivariate analysis of covariance (for DASS subscales) that were performed using SPSS/21 software. 

## RESULTS

Demographic variables in the intervention and control groups are reported in Table 1. The mean age of the subjects was 47.45±9.15 and 47.73±9.15 years in the intervention and control groups respectively. According to Chi-squared test, the two groups were similar regarding demographic variables including sex (P=0.78), educational level (P=0.76), frequency of patients’ hospitalization (P=0.81), and MI events (P=0.39) (Table 1).

**Table 1:** Demographic variables in the intervention (n=40) and control (n=38) groups

Variable	Intervention Group	Control Group	P value*
	N (%)	N (%)	
Educational level			0.76
Illiterate	3 (7.50)	5 (13.20)	
Elementary or secondary education	22 (55)	19 (50)	
High school diploma	7 (17.50)	5 (13.20)	
Post-secondary education	8 (20)	9 (23.70)	
Number of patients’ hospitalization			0.81
Once	29 (72.50)	29 (76.30)	
Twice	6 (15)	5 (13.20)	
More than twice	5 (12.50)	4 (10.50)	
Number of MI^a^ events			0.39
Once	34 (85)	28 (73.70)	
Twice	5 (12.50)	9 (23.70)	
More than twice	1 (2.50)	1 (2.60)	
Sex			0.78
Women	34 (85)	33 (86.80)	
Men	6 (15)	5 (13.20)	

* Chi-squared test;

a Myocardial Infarction

Table 2 presents the mean and standard deviation of the perceived control and DASS scores. Furthermore, in this Table the result of paired sample t-test and independent sample t-test is presented. As we can see, the scores of perceived control (P=0.04), depression (P=0.002), anxiety (P=0.001) and stress (P=0.006) decreased significantly from the pretest to posttest in the intervention group. However, in the control group the scores of perceived control (P=0.001), depression (P=0.001), anxiety (P=0.03) and stress (P=0.001) increased significantly from pretest to posttest. 

**Table 2:** The mean, standard deviation and paired sample t test for comparing the means of perceived control, and DASS subscales in the intervention and control groups

Variable	Group	Time	Score (mean±SD)	P value*	P value**
Perceived Control	Intervention	Pre Test	9.42±3.33	P=0.04	P=0.001
		Post Test	8.15±3.65		
	Control	Pre Test	9.28±4.44	P=0.001	
		Post Test	12.15±4.88		
Depression	Intervention	Pre Test	28.85±11.99	P=0.002	P=0.01
		Post Test	21.65±8.64		
	Control	Pre Test	23.05±6.78	P=0.001	
		Post Test	26.02±7.64		
Anxiety	Intervention	Pre Test	26.67±9.46	P=0.001	P=0.003
		Post Test	20.70±6.29		
	Control	Pre Test	23.26±6.37	P=0.03	
		Post Test	24.36±7.12		
Stress	Intervention	Pre Test	30.20±10.91	P=0.006	P=0.04
		Post Test	24.32±10.23		
	Control	Pre Test	25.86±6.85	P=0.001	
		Post Test	30.23±6.90		

* Paired sample t test for within group comparison;

** Independent sample t test for between group comparison in posttest

To examine the effect of CPR and cardiac chest pain management training on Perceived Control, Depression, Stress and Anxiety, we used the analysis of covariance, so that the subjects’ pretest and posttest scores were used as covariate and dependent variables, respectively and the intervention variables were used as independent variable. The result of one way analysis of covariance for examination of the effectiveness of CPR and Cardiac Chest Pain Management Trainings on Perceived Control is presented in Table 3. 

**Table 3:** The result of one way analysis of covariance for the effectiveness of intervention on the subjects’ perceived control

Source of Variance	Sum of Squares	DF	Mean Square	F	P value*
Pretest (Perceived Control)	816.83	1	817	175.65	0.0001
Intervention	68.56	1	69	14.74	0.0001
Within Groups	348.76	75	5		
Total	1165.95	77			

* One way analysis of covariance

As shown in Table 3, after controlling for the preexisting differences between the intervention and control groups (scores of perceived control in pretest administration), there was a significant difference (P<0.0001) between the two groups (intervention vs. control) in the perceived control scores in posttest administration. Based on descriptive statistics displayed in Table 2, it can be said that the intervention decreased the scores of perceived control in the intervention group. This low score represents an internal locus of control in the spouses of the intervention group. That means, the sense of perceived control in the spouses of this group has increased significantly after the intervention. The result of multivariate analysis of covariance for examination of the effectiveness of CPR and Cardiac Chest Pain Management Trainings on depression, stress and anxiety (as three related subscale) is presented in Table 4.

**Table 4:** The result of multivariate analysis of covariance for the effectiveness of the intervention on the subjects’ depression, stress and anxiety

Source of Variance	Dependent variables	Sum of Squares	DF	Mean Square	F	P value*
Pretest (DASS)	Depression	4670.69	1	4671	132.37	0.0001
	Anxiety	3313.85	1	3314	147.39	0.0001
	Stress	4075.67	1	4076	132.49	0.0001
Intervention	Depression	452.16	1	452	12.81	0.001
	Anxiety	130.61	1	131	5.81	0.02
	Stress	227.65	1	228	7.40	0.01
Within Groups	Depression	2646.31	75	35		
	Anxiety	1686.29	75	22		
	Stress	2307.08	75	31		
Total	Depression	7971.95	77			
	Anxiety	5226.99	77			
	Stress	6748.37	77			

* The multivariate analysis of covariance

As seen in Table 4, after controlling for the preexisting differences between the intervention and control groups (total scores of DASS in pretest administration), there was a significant difference between the two groups (intervention vs. control) in the scores of depression (P<0.001), anxiety (P=0.02) and stress (P=0.01) in posttest administration. Based on the descriptive statistics shown in Table 2, it can be said that the intervention has lowered the scores of depression, stress and anxiety in experimental group. 

## DISCUSSION

According to the results, it can be concluded that CPR trainings and chest pain management has a positive effect on the level of anxiety in the spouses of the patients with MI, reducing it in the intervention group. One reason for this decrease can be the presence of telephone follow-up, contact with spouses and answering their questions about the training during the study.

The results of the present study reported that the mean scores of perceived control significantly decreased after the intervention in the intervention group. This decline indicated the positive effects of training CPR and chest pain management for spouses in the intervention group. Decreased level of perceived control score in the intervention group represents the inner perceived control orientation and increases the sense of perceived control in the spouses of the intervention group after the training. Other studies have shown that CPR training and chest pain management can also increase the spouses’ knowledge, and such an increase is one of the important factors in increasing internal perceived control. ^9 , 19^ Providing information can reduce the psychological stresses by enhancing perceived control. ^8 , 9 , 26^ So far, the studies conducted in Iran on perceived control were on asthma patients ^28^ and students, ^27^ indicating the impact of education on internalization of the perceived control. Considering the matching results of other studies on other groups, we should take this point into account in managing emergency situations in the family of heart patients.

The present study showed that the anxiety scores in the intervention group improved after the intervention. Previous studies for caregivers of hospitalized patients ^29^ and mentally retarded children’s parents ^30^ provide evidence that supportive educational interventions such as CPR trainings and social supports could reduce anxiety. A study was conducted on diabetic patients showed that coping skills training reduced anxiety. ^20^ Another study also demonstrated that the counseling and training of the spouses as a simple intervention can reduce anxiety. ^8^

Our results also confirmed that the participants of both groups experienced the severe and extremely severe level of depression before the intervention. However, the depression mean scores in the intervention group decreased after six weeks. This reflects the significant effect of training on the depression level of spouses in such groups. Other researchers in Iran have proven that consultation and trainings could reduce the depression level in mentally retarded children’s parents, ^30^ family caregivers of patients with cancer, ^31^ and diabetic patients. ^20^ Studies have also shown that anxiety and depression reduction in spouses may also be a cost-effective way to improve the psychosocial status, decrease mortality and morbidity in the patient groups. ^8^ Therefore, considering the important role of education in reducing anxiety and depression, it is essential that educating the spouses and family members of patients is considered as an important topic in the curative treatment program, especially for nurses.

We observed that stress mean scores significantly decreased in the intervention group after the intervention. This confirms the significant effect of CPR and chest pain management trainings on the stress level of spouses. Many similar studies on other disease (mentally retarded children’s parents, ^30^ and cancer ^31^ ) confirmed the significant effects of consultation and trainings on reduction of stress.

On the other hand, the results showed that the level of depression, anxiety, and stress in the control group not only did not decrease, but also actually increased after six weeks. Such an increase can result from stressful conditions of the spouses after MI. Also, psychological stress often continues months after a heart attack. ^8 , 12^ Lack of sufficient knowledge about cardiac chest pain management and lack of skills to perform CPR and take appropriate actions in cardiac emergencies are considered as other possible causes. ^8^ It is expected that such training can increase the spouses’ abilities to support their patients.

Studies with longer follow-up periods are recommended to investigate the prolonged effects of CPR training and chest pain management on improvement of the perceived control, depression, anxiety, and stress in spouses.

## CONCLUSION

Considering the importance and effects of basic CPR training and chest pain management to the spouses of patients with MI, the managers of medical and healthcare systems should plan to provide such services for the patients’ families. The role of nurses in implementing and promoting such programs should also be considered. Accordingly, not only their educational needs will be met, the patients’ families will also receive supports. Furthermore, we can hope to achieve positive outcomes for the patients with MI in this regard. The Ministry of Health and Medical Education should conduct a training session on how to deal with heart attack patients practically at the community level (schools, offices, health centers, etc.). The outcomes were evaluated only six weeks after the educational intervention. Thus, future studies with longer follow-up periods are recommended for better evaluation. The low sample size of the participants was another limitation of the study.

Pain management and CPR trainings could effectively increase the perceived internal control and decrease depression, stress, and anxiety in the spouses of patients with MI. Considering the importance and positive effects of CPR training, we can use in rehabilitation and training programs for the patients and their families. The role of nurses in implementing and promoting Cardiac Chest Pain Management and CPR Trainings should be considered.
